# Exploiting gender-based biomarkers and drug targets: advancing personalized therapeutic strategies in hepatocellular carcinoma

**DOI:** 10.3389/fphar.2024.1433540

**Published:** 2024-06-20

**Authors:** Lanqian Su, Huanyu Luo, Yalan Yan, Zhongqiu Yang, Jiaan Lu, Danqi Xu, Linjuan Du, Jie Liu, Guanhu Yang, Hao Chi

**Affiliations:** ^1^ Clinical Medical College, Southwest Medical University, Luzhou, China; ^2^ Department of General Surgery, Dazhou Central Hospital, Dazhou, China; ^3^ Department of Oncology, Dazhou Central Hospital, Dazhou, China; ^4^ Department of Specialty Medicine, Ohio University, Athens, OH, United States

**Keywords:** hepatocellular carcinoma, gender heterogeneity, gender-specific therapies, cancer immunotherapy, sex hormone, molecular pathways, drug targets, treatment strategies

## Abstract

This review systematically examines gender differences in hepatocellular carcinoma (HCC), identifying the influence of sex hormones, genetic variance, and environmental factors on the disease’s epidemiology and treatment outcomes. Recognizing the liver as a sexually dimorphic organ, we highlight how gender-specific risk factors, such as alcohol consumption and obesity, contribute differently to hepatocarcinogenesis in men and women. We explore molecular mechanisms, including the differential expression of androgen and estrogen receptors, which mediate diverse pathways in tumor biology such as cell proliferation, apoptosis, and DNA repair. Our analysis underscores the critical need for gender-specific research in liver cancer, from molecular studies to clinical trials, to improve diagnostic accuracy and therapeutic effectiveness. By incorporating a gender perspective into all facets of liver cancer research, we advocate for a more precise and personalized approach to cancer treatment that acknowledges gender as a significant factor in both the progression of HCC and its response to treatment. This review aims to foster a deeper understanding of the biological and molecular bases of gender differences in HCC and to promote the development of tailored interventions that enhance outcomes for all patients.

## 1 Introduction

Globally, liver cancer constitutes the third-highest cancer mortality, with approximately 90% through Hepatocellular carcinoma (HCC) (Kuwano et al., 2022). According to the GLOBOCAN 2020 database survey, it was estimated that about 9.5 and 8.7 ratios of age-standardized new cases and deaths in the world accounted for liver cancer, respectively, which has been increasing (Wei et al., 2014). Currently, the tumor has been treated with surgical resection, liver transplantation, chemotherapy, radiotherapy, and targeted therapies such as sorafenib (Jiang et al., 2019; Qi et al., 2020; Li et al., 2023a; Su et al., 2023a; Zhang S. et al., 2023). While surgery and transplantation can be done in the early stage of the disease, however, most of the patients are diagnosed at a later age of the tumor, where the tumor has advanced and cannot be amenable to surgery and transplantation (Pan et al., 2014; Chaoul et al., 2020; Su et al., 2022a). Chemotherapy and radiotherapy treatments are characterized by systemic toxicity and side effects, but the so-called targeted treatment is emerging and in advanced stages, it is already very promising, although it still encounters the problem of drug resistance and a high relapse rate (Kamimura et al., 2020; Su et al., 2022b; Li et al., 2023b; Su et al., 2023b; Gao et al., 2023). This really highlights the urgent need for advances in early detection and more effective systemic therapies that are individualized and take into account patient differences at all levels, including gender (Chi et al., 2023; Grani et al., 2023).

The liver is highly sexually dimorphic, and a combination of hormonal, genetic, and environmental factors greatly influence the gender differences observed in hepatocarcinogenesis, treatment, and incidence (Marker et al., 2023; Huillet et al., 2024). For instance, the liver is very sensitive to sex hormones that include androgens and estrogens, and differences in molecular pathways have been noticed during the hepatocarcinogenesis phase, such as gene expression associated with the regulation of the cell cycle, apoptosis, and DNA repair (Singhal and Schlondorff, 1987; LoMauro and Aliverti, 2021). The liver is a tissue that bears additional sex-specific risk factors, one being alcohol consumption and obesity for the development of HCC. Some sex-specific risk factors, including alcohol intake, obesity, and insulin resistance, have been implicated in hepatocarcinogenesis, likely due to sex differences in alcohol metabolism and fat distribution impacting susceptibility to HCC (D’Souza et al., 2020; Izquierdo et al., 2022; Kardashian et al., 2023). That highlights the pressing need for a transition to a gender perspective in the entire flow of liver cancer research, from epidemiological inquiry to molecular analysis.

Here, we have presented a systematic review of several dimensions of the impact of gender differences on HCC, including the genetic background of the disease, pathogenesis, treatment response, and prognosis. The aim is to promote a more precise medical approach, leading to better outcomes for all patients with liver cancer (Nan et al., 2021).

## 2 Factors affecting gender differences in HCC

The proposed mechanisms for gender differences in HCC are thought to be complex and multifactorial (Pok et al., 2016). They are currently attributed to gender differences in environmental objective factors, behavioral risk factors, immune responses, metabolic risk factors, tumor biology and hormonal factors (Bashir Hamidu et al., 2021).

### 2.1 Environmental and lifestyle factors

Geographical differences in HCC and its etiology are clear; in general, they are due to the distribution of risk factors and different development between regions (Mousavi et al., 2013; Yan et al., 2020; Enomoto et al., 2021). Indeed, the highest age-standardized incidence rates (ASRs) of HCC are estimated in East Asia, North Africa, and South-East Asia (Jiang et al., 2012; Okoronkwo et al., 2017). Sex differences are also reflected in the risk factors of HCC: Studies in recent years demonstrate that HBV and HCV are the major infectious agents associated with liver cancer (Zhang et al., 2020; Wang et al., 2021). The prevalence of HBV infection is greater among males than females (Poorolajal and Majdzadeh, 2009). However, the incidence of HCV is higher among females at 20.36 cases per 100 person-years than among males at 15.20 cases per 100 person-years (Puri et al., 2014). These days, the impact of viral hepatitis on liver cancer is waning, due to effective therapies (HBV, HCV) and vaccines (Nasr et al., 2023).

Non-viral causes (especially heavy consumption of alcohol) appear to have partially replaced the role of diseases caused virally in the case of HCC (Lee et al., 2021; Zhang J. et al., 2023). Effects of alcohol and its metabolites vary with age, race, and gender, with gender being marked mostly by differences. In terms of alcohol metabolism and in the context of heavy drinking, the relationship with HCC in women is stronger than in men, perhaps due to higher activities of alcohol dehydrogenases in women or a more prominent link of alcohol intake with cirrhosis risk in women (Bell et al., 2004; KASL, 2012). In meta-analyses, heavy drinking (≥4 drinks/day) was associated with about a fourfold risk for women but only about a 59% increase for men (McGlynn et al., 2021). However, a higher intake of alcohol by men is experienced than women (Milman and Kirchhoff, 1996). Although heavy alcohol drinking has been established as one of the risk factors for liver cancer, most data indicated a weak negative association with light or moderate alcohol drinking and a reduced risk of HCC (Gao et al., 2020; Liu et al., 2022; Singh et al., 2023).

### 2.2 Inheritance and gene expression

**FIGURE 1:**
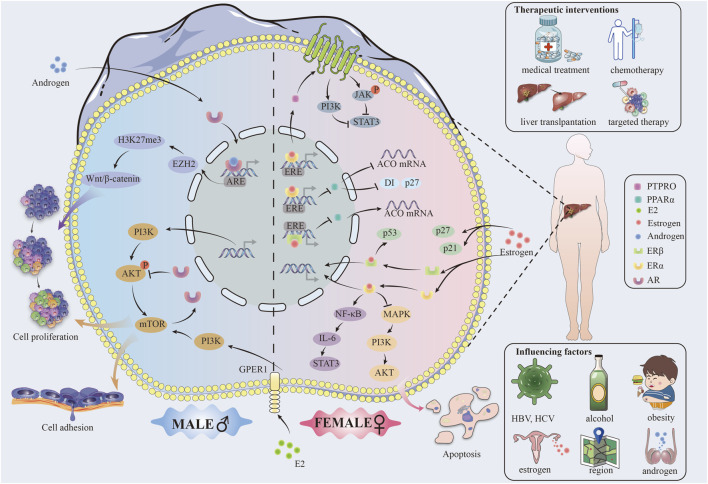
Gender differences in hepatocellular cancer.

Males and females present an active difference in gene expression. Figure 1 For example, studies have demonstrated that in male hepatocytes derived from individuals with HCC, the androgen receptor (AR) significantly enhances the expression of Enhancer of zeste homolog 2 (EZH2) at the transcriptional level. This enhancement facilitates an increase in the trimethylation level at lysine 27 of histone H3 (H3K27me3), effectively repressing the inhibitors of Wnt signaling pathways. This event activates Wnt/β-cyclin signaling and promotes the proliferation and transformation of liver tumor cells (Tsang et al., 2016; Baliou et al., 2020). On the other hand, estrogen in females, acting through the ERα receptor, can upregulate the protein tyrosine phosphatase receptor type O (PTPRO), which serves as a wide spectrum of cancer types (HCC, colorectal carcinoma, etc.) tumor suppressor protein (Asbagh et al., 2014; Xu et al., 2014).α binds to the estrogen response element (ERE) of the PTPRO gene promoter, inducing dephosphorylation of Janus kinase 2 (JAK2) and phosphatidylinositol 3-kinase (PI3K), which in turn causes a decrease of the activity of the transcription factor STAT3, thus leading to inhibition of the HCC cell proliferation (Wu and Lou, 2023; Su et al., 2024).

Moreover, Erα binds directly to the ERE of the peroxisome proliferator-activated receptor alpha (PPARα) gene, which is a nuclear receptor protein with the function of a transcription factor, crucial for the oxidative processes in the hepatocytes (Memaj et al., 2023). Together, they decrease transcription of the PPARα gene and further regulate PPARα target acyl-coenzyme A oxidase (ACO), cell cycle proteins D1 and P27, blocking the proliferation of cancer cells and promoting apoptosis (Meng and Liu, 2022).

ERβ can downregulate PPARα and its downstream genes through interaction with the EREs of the PPARα gene to inhibit HCC development (Meng and Liu, 2022). In addition, it is through the action of ERβ that the translocation of PPARα from the cytoplasm to the nucleus is prevented, and the transcription activity of PPARα consequently decreases. This hormone-receptor complex subsequently induces homodimerization or heterodimerization of ER, translocation to the nucleus, binding to EREs on promoters of target genes, and induction of genomic effects of gene activation and epigenetic changes (Krolick and Shi, 2022).

In order to gain a comprehensive understanding of the factors influencing sex differences, it is essential to consider the regulatory networks downstream of hormones, in addition to genetic factors and the direct role of sex hormones. Estrogens can indirectly bring about the expression of genes by interaction with specific transcription factors through non-genomic effects, which can influence signaling pathways for the development of HCC (Mandalà, 2020). For example, ERα interacts with the repressor NF-κB by inhibiting the IL-6/STAT3 activation pathway (Meng and Liu, 2022).

### 2.3 Influence of sex hormone

Estrogen and androgen have a key role in the molecular mechanisms of HCC (Liu et al., 2020). Estrogen can block the production of IL-6, a pro-inflammatory tumor growth and metastasis-promoting factor, through the JAK/STAT signaling pathway. At the same time, estrogen decreases the expression of TNF-α, another pro-inflammatory cytokine able to activate cancer cells through the NF-κB signaling pathway (Miller, 2018). On the other side, androgens could further enhance the development of HCC through increased expression of the above pro-inflammatory cytokines, exaggerating the inflammatory response (Wu et al., 2015). Androgens also have been demonstrated to upregulate the proliferation of HCC cells by activating their receptor AR, which in turn promotes the expression of c-Myc, an important regulator of cell proliferation and survival (Bao et al., 2020; Cho et al., 2020; Cui et al., 2020; Zhu et al., 2020; Gao et al., 2021) (Supplementary Table S1).

## 3 Molecular mechanisms and gender differences in HCC

### 3.1 Mechanisms of proliferation, invasion and metastasis

In HCC, gender differences have profound effects on tumor cell proliferation, invasion, and metastasis, where mechanistic target of rapamycin (mTOR) signaling is associated with many features of cancer (Ferrín et al., 2020). (FIGURE)On the one hand, AR negatively regulates the feedback activation of AKT-mTOR signaling (Zhang et al., 2018). On the other hand, mTOR promotes the expression of nuclear AR protein by inhibiting ubiquitin-dependent AR degradation and enhancing its nuclear localization through enhancing the nuclear localization of AR, consequently mechanistically explaining AR overexpression in the nucleus of HCC cells (Zhang et al., 2018). AR overexpression was strongly associated with advanced tumor stage and low survival29220539. Approximately a third of HCC tumors showed overexpressed nuclear AR protein in a series of 142 paired HCC tumors and their neighboring non-cancerous liver tissues (Zhang et al., 2018).

Furthermore, research has demonstrated that the estrogen receptor complex inhibits the mTOR signaling pathway, thereby impeding tumor growth (Ke et al., 2022). The activation of the PI3K-Akt (∼70%) and mechanistic target of rapamycin complex 1 (mTORC1) (∼45%) pathways was observed in HCC and demonstrated a positive correlation with tumor metastasis, recurrence and poor prognosis (Chaturantabut et al., 2019). A study using the HCC zebrafish model suggested that G protein-coupled estrogen receptor 1 (GPER1) could be a factor in the progress of hepatocarcinogenesis by inducing proliferation of hepatocytes and regulating organ growth via GPER1-PI3K-mTOR signaling transduction (Wojnarowski et al., 2022). E2–The pro-proliferative consequences of PI3K-mTOR signaling activation by GPER1 and the strong response to the presence of GPER1 antagonist therapy during cancer development and progression, as evidenced by *in vivo* human data (Ferrín et al., 2020; Tian et al., 2023). All these experimental results point to the fact that drugs targeted at E2-GPER1 should offer a new promising application for therapeutic use in liver cancer prevention and treatment (Chaturantabut et al., 2019).

Activation of the PI3K/AKT signaling pathway promotes hepatocyte proliferation and increases the capability of epithelial mesenchymal transition (EMT) through increasing HCC cell growth, migration, and invasion (Cantile et al., 2019). AR upregulates integrin β1 expression through the PI3K/AKT/mTOR signal pathway, consequently, increasing in cellular adhesion, which could be a potential characteristic of advanced hepatocellular cancer with high metastasis (Carlos-Reyes et al., 2021). However, it was found that mice lacking hepatic AR developed more undifferentiated tumors and larger tumor sizes at the late metastatic stage compared to mouse models expressing AR, and these mice also died earlier due to increased lung metastasis. This suggests that hepatic AR may play a dual but opposing role in promoting HCC development and inhibiting HCC metastasis (Ma et al., 2012; Wen et al., 2014).

Studies have indicated that there should be gender specificity of p53 gene mutations in the development process of HCC (Shi et al., 1995). In addition, mutations in p53, a key oncogene for cell cycle regulation and apoptosis, were seen to hasten tumor progression (Chuery et al., 2017). Men suffering from liver cancer were more associated with the frequency of p53 mutations than women (Finch and Tower, 2014). Besides, p53 is a vital regulator for the cellular response to DNA damage (Li and Wong, 2018). The ERβ complex in estrogen (ERβ) partially contributes to the stabilization and activation of p53 in HCC cells, thus prohibiting the delivery of damaged DNA through aberrant cell cycle arrest and apoptosis.

### 3.2 Cell cycle regulation and apoptosis

Sex differences exert their influence on cell cycle regulation through the alteration of key regulatory proteins such as cyclins, cyclin-dependent kinases (CDKs), and CDK inhibitors, including p21 and p27 (Lim and Kaldis, 2013). (FIGURE) Estrogens can upregulate the expression of p21 and p27, which will lead to cell arrest in phase G1 by stopping the activity of CDK, blocking the tumor cell cycle (Eto, 2010; Madhu Krishna et al., 2018). On the other hand, androgens downregulate the expression of these inhibitory proteins, thereby bringing the cell cycle on and causing tumor proliferation (Yu et al., 2017).

Apoptosis is programmed cell death, a process that assumes huge importance as a self-regulatory mechanism in the organism (Li et al., 2019). The identified Bcl-2 family proteins to date have an anti-apoptotic function, for example, Bcl-2, and pro-apoptotic action, for example, Bax (Chen et al., 2016). Estrogens will increase the expression of pro-apoptotic proteins, including Bax, to promote programmed cell death in damaged cells, whereas androgens may support the intensification of survival signaling, for example, by increasing the expression of Bcl-2 proteins that inhibit apoptosis (Arunkumar et al., 2012; Herson et al., 2013; Sanaei et al., 2022).

## 4 Medical treatment in HCC

Studies carried out in the tumor microenvironment (TME), immune response, Liver Transplant (LT) acceptance rate, and hormone therapy have alluded to a significant effect of gender differences on the outcomes of cancer treatment and survival. This fact propounds that future treatment strategies can incorporate gender-specific immune response and hormone modulation for more precise and effective anti-cancer strategies.

Sexual dimorphism exists in the immune response (Rehman and Masson, 2005; Wu et al., 2009; Mitchell et al., 2020). Women generate both adaptive and innate immunity responses much stronger than men, but at the same time, they suffer from systemic autoimmune diseases much more highly than men (Murgia et al., 2022; Zhang et al., 2022). In the case of non-small cell lung cancer (NSCLC) at an early stage, males present with a cold TME in which there is a defect in T-cell rejection. Contrary to that, female patients have a hotter TME, greater infiltration of dendritic cells (DCs), CD8 T cells and CD4 T cells, and greater upregulation of immune checkpoint molecules in T cells (Conforti et al., 2021).

In advanced HCC, liver transplantation is the standard treatment for end-stage liver disease (ESLD) (Hill et al., 2023). Studies have shown that women are less likely than men to receive LT because the hypothesis responsible for the gender-based variation in radical treatment is limited by the ability of Model for End-Stage Liver Disease (MELD) scores based on cr measurements in females (Mindikoglu et al., 2013; Karnam et al., 2021). In one study, females received 1–2.4 fewer cr-derived MELD scores compared to males with similar renal function (Allen et al., 2018). However, researchers came up with a new multivariate model, MELD 3.0, meant to account for the factor of gender difference on waiting lists (Kim et al., 2021).

Sex differences in treatments that antagonize sex hormones and sex hormone receptors. Anti-ER therapy was found to promote tumor development in a mouse model, however, several studies have demonstrated that anti-AR therapy inhibits liver tumorigenesis (Ahotupa et al., 1994; Williams et al., 1997; Ma et al., 2012; Tang et al., 2021). Anti-hormonal therapy primarily disrupts the interaction between hormones and hormone receptors, thereby modulating downstream targets. However, the effect of anti-hormone therapy on HCC has been controversial. Very few clinical studies or randomized control trials demonstrate increased survival or survival in patients with advanced HCC. Most of the studies concluded that patients with HCC do not benefit from antihormonal drug therapy, mainly from side effects from the drugs and variability of the estrogen receptor. Survival outcomes in patients with HCC are affected by gender differences (Farinati et al., 1990; Martínez Cerezo et al., 1994; Grimaldi et al., 1998; Li Z. et al., 2023).

Gender differences affect survival outcomes in patients with HCC.HBV-infected male patients have an increased incidence of HCC compared to women, while men have higher serum HBV DNA titres. These data suggest that the overall survival among men is significantly shortened in comparison to women among patients with HCC (Chen et al., 2009; Sayaf et al., 2022). Female HBV patients have a decreased risk for HCC and improved survival with hormone replacement therapy (HRT) (Hassan et al., 2017; Wang et al., 2022). Ten thousand four hundred seventy-four women in the cohort study were postmenopausal and infected with HBV. Incidence rate in the HRT group of HCC and all-cause mortality of the HRT group decreased, compared with those in the no HRT group. Indeed, parallel research has concluded that an association exists between HRT and reduced HCC risk and better survival outcomes (Wang et al., 2022).

## 5 Discussion

The liver is a highly sexually dimorphic organ, possessing at least 72% of sexually differentiated genes (Yang et al., 2006). Sex hormones play a central role in gender preference in HCC, and thus multiple anti-sex hormone therapies or anti-sex hormone receptor therapies have been tried. Tamoxifen (TMX) therapy and hormone replacement therapy (HRT) are the two core regimens for hormone therapy in HCC (Meng and Liu, 2022).Although the efficacy of TMX in HCC remains controversial, there are still relevant studies reporting a positive relationship between the cancer inhibitory effect of TMX and ERα expression levels. In the work of Villa et al., 50 HCC patients were differentiated by wild-type ERα and ER α mRNA variant lacking exon 5 (ERΔ5) phenotypes and the therapeutic efficacy of TMX was confirmed in patients with wild-type phenotype (Villa et al., 1996). Thereby the use of hormone therapy may largely dependent on the classification of ERα and screen or amplification of HCC patients with higher ERα expression may be beneficial to improve the sensitivity of hormone therapy. The effectiveness of estrogen replacement therapy in HCC has been demonstrated to some extent, however, estrogen may increase the risk of breast, ovarian and endometrial cancer in female patients and may have an unfavorable effects (American Medical Association, 2002; Meng and Liu, 2022; Wang et al., 2022). Exploring HCC hormone therapy in combination with first-line drugs may be an option to improve efficacy.

AR is a crucial player in male dominant hepatocarcinogenesis. On one hand, abundant evidence shows that androgens exert tumor-promoting effects. On the other hand, AR blockade has been proved to do little benefit for HCC patients. It may be a fact that differences in sex hormone profiles are important not only in the initiation but also at the different stages of hepatocarcinogenesis, for example, the anti-tumor functions of AR in metastatic HCC (Ma et al., 2012). In addition, AR overexpression might also be used as an independent factor to predict the prognosis of patients with HCC. However, a portion of HCC was detected with the expression of C-terminal truncated AR-SVs. AR-SVs have been identified to play an important role in the acquired resistance to AR inhibitors (Dauki et al., 2020; Qiao et al., 2021; Katleba et al., 2023). Therefore, we imply that AR-SVs might also be involved in the occurrence of acquired resistance to AR inhibitors in HCC.

## References

[B1] Ahotupa M. Hirsimäki P. Pärssinen R. Mäntylä E. (1994). Alterations of drug metabolizing and antioxidant enzyme activities during tamoxifen-induced hepatocarcinogenesis in the rat. Carcinogenesis 15, 863–868. 10.1093/carcin/15.5.863 8200088

[B2] Allen A. M. Heimbach J. K. Larson J. J. Mara K. C. Kim W. R. Kamath P. S. (2018). Reduced access to liver transplantation in women: role of height, MELD exception scores, and renal function underestimation. Transplantation 102, 1710–1716. 10.1097/TP.0000000000002196 29620614 PMC6153066

[B3] American Medical Association (2002). Long-term use of estrogen-only hormone replacement therapy (HRT) linked with increased risk of ovarian cancer. Ginecol. Obstet. Mex. 70, 409–410. 12448047

[B4] Arunkumar R. Sharmila G. Elumalai P. Senthilkumar K. Banudevi S. Gunadharini D. N. (2012). Effect of diallyl disulfide on insulin-like growth factor signaling molecules involved in cell survival and proliferation of human prostate cancer cells *in vitro* and *in silico* approach through docking analysis. Phytomedicine 19, 912–923. 10.1016/j.phymed.2012.04.009 22739413

[B5] Asbagh L. A. Vazquez I. Vecchione L. Budinska E. De Vriendt V. Baietti M. F. (2014). The tyrosine phosphatase PTPRO sensitizes colon cancer cells to anti-EGFR therapy through activation of SRC-mediated EGFR signaling. Oncotarget 5, 10070–10083. 10.18632/oncotarget.2458 25301722 PMC4259406

[B6] Baliou S. Kyriakopoulos A. M. Spandidos D. A. Zoumpourlis V. (2020). Role of taurine, its haloamines and its lncRNA TUG1 in both inflammation and cancer progression. On the road to therapeutics? (Review). Int. J. Oncol. 57, 631–664. 10.3892/ijo.2020.5100 32705269 PMC7384849

[B7] Bao S. X. Wang C. H. Jin S. Hu K. W. Lu J. T. (2020). miR-135b-5p suppresses androgen receptor-enhanced hepatocellular carcinoma cell proliferation via regulating the HIF-2α/c-Myc/P27 signals *in vitro* . Onco Targets Ther. 13, 9991–10000. 10.2147/OTT.S268214 33116584 PMC7548343

[B8] Bashir Hamidu R. Chalikonda D. M. Hann H. W. (2021). Gender disparity in host responses to hepatitis B-related hepatocellular carcinoma: a case series. Vaccines (Basel) 9, 838. 10.3390/vaccines9080838 34451963 PMC8402514

[B9] Bell B. P. Mast E. E. Terrault N. Hutin Y. J. (2004). Prevention of hepatitis C in women. Emerg. Infect. Dis. 10, 2035–2036. 10.3201/eid1011.040624_04 16010740 PMC3329031

[B10] Cantile M. Palmieri G. Botti G. (2019). Developmental gene markers in tumor pathogenesis and progression. Dis. Markers 2019, 5462562. 10.1155/2019/5462562 31360264 PMC6644247

[B11] Carlos-Reyes A. Muñiz-Lino M. A. Romero-Garcia S. López-Camarillo C. Hernández-de la Cruz O. N. (2021). Biological adaptations of tumor cells to radiation therapy. Front. Oncol. 11, 718636. 10.3389/fonc.2021.718636 34900673 PMC8652287

[B12] Chang-Lee S. N. Hsu H. H. Shibu M. A. Ho T. J. Tsai C. H. Chen M. C. (2017). E2/ERβ inhibits PPARα to regulate cell-proliferation and enhance apoptosis in Hep3B-hepatocellular carcinoma. Pathol. Oncol. Res. 23, 477–485. 10.1007/s12253-016-0136-8 27757837

[B13] Chaoul N. Mancarella S. Lupo L. Giannelli G. Dituri F. (2020). Impaired anti-tumor T cell response in hepatocellular carcinoma. Cancers (Basel) 12, 627. 10.3390/cancers12030627 32182707 PMC7139707

[B14] Chaturantabut S. Shwartz A. Evason K. J. Cox A. G. Labella K. Schepers A. G. (2019). Estrogen activation of G-protein-coupled estrogen receptor 1 regulates phosphoinositide 3-kinase and mTOR signaling to promote liver growth in zebrafish and proliferation of human hepatocytes. Gastroenterology 156, 1788–1804. 10.1053/j.gastro.2019.01.010 30641053 PMC6532055

[B15] Chen C. J. Yang H. I. Iloeje U. H. (2009). Hepatitis B virus DNA levels and outcomes in chronic hepatitis B. Hepatology 49, S72–S84. 10.1002/hep.22884 19399801

[B16] Chen M. Wu J. Luo Q. Mo S. Lyu Y. Wei Y. (2016). The anticancer properties of herba epimedii and its main bioactive componentsicariin and icariside II. Nutrients 8, 563. 10.3390/nu8090563 27649234 PMC5037548

[B17] Chi H. Zhao S. Yang J. Gao X. Peng G. Zhang J. (2023). T-cell exhaustion signatures characterize the immune landscape and predict HCC prognosis via integrating single-cell RNA-seq and bulk RNA-sequencing. Front. Immunol. 14, 1137025. 10.3389/fimmu.2023.1137025 37006257 PMC10050519

[B18] Cho Y. Park M. J. Kim K. Kim S. W. Kim W. Oh S. (2020). Reactive oxygen species-induced activation of Yes-associated protein-1 through the c-Myc pathway is a therapeutic target in hepatocellular carcinoma. World J. Gastroenterol. 26, 6599–6613. 10.3748/wjg.v26.i42.6599 33268949 PMC7673967

[B19] Chuery A. C. S. Silva I. Ribalta J. C. L. Speck N. M. G. (2017). Association between the p53 arginine/arginine homozygous genotype at codon 72 and human papillomavirus E6/E7 mRNA expression. Braz J. Infect. Dis. 21, 248–254. 10.1016/j.bjid.2017.03.002 28347732 PMC9428040

[B20] Conforti F. Pala L. Pagan E. Bagnardi V. De Pas T. Queirolo P. (2021). Sex-based dimorphism of anticancer immune response and molecular mechanisms of immune evasion. Clin. Cancer Res. 27, 4311–4324. 10.1158/1078-0432.CCR-21-0136 34016641 PMC7611463

[B21] Cui S. Z. Lei Z. Y. Guan T. P. Fan L. L. Li Y. Q. Geng X. Y. (2020). Targeting USP1-dependent KDM4A protein stability as a potential prostate cancer therapy. Cancer Sci. 111, 1567–1581. 10.1111/cas.14375 32133742 PMC7226285

[B22] Dauki A. M. Blachly J. S. Kautto E. A. Ezzat S. Abdel-Rahman M. H. Coss C. C. (2020). Transcriptionally active androgen receptor splice variants promote hepatocellular carcinoma progression. Cancer Res. 80, 561–575. 10.1158/0008-5472.CAN-19-1117 31685543 PMC7002251

[B23] D’Souza S. Lau K. C. Coffin C. S. Patel T. R. (2020). Molecular mechanisms of viral hepatitis induced hepatocellular carcinoma. World J. Gastroenterol. 26, 5759–5783. 10.3748/wjg.v26.i38.5759 33132633 PMC7579760

[B24] Enomoto H. Ueno Y. Hiasa Y. Nishikawa H. Hige S. Takikawa Y. (2021). The transition in the etiologies of hepatocellular carcinoma-complicated liver cirrhosis in a nationwide survey of Japan. J. Gastroenterol. 56, 158–167. 10.1007/s00535-020-01748-x 33219410 PMC7862502

[B25] Eto I. (2010). Upstream molecular signaling pathways of p27(Kip1) expression: effects of 4-hydroxytamoxifen, dexamethasone, and retinoic acids. Cancer Cell Int. 10, 3. 10.1186/1475-2867-10-3 20170512 PMC2841156

[B26] Farinati F. Salvagnini M. de Maria N. Fornasiero A. Chiaramonte M. Rossaro L. (1990). Unresectable hepatocellular carcinoma: a prospective controlled trial with tamoxifen. J. Hepatol. 11, 297–301. 10.1016/0168-8278(90)90211-9 1705274

[B27] Ferrín G. Guerrero M. Amado V. Rodríguez-Perálvarez M. De la Mata M. (2020). Activation of mTOR signaling pathway in hepatocellular carcinoma. Int. J. Mol. Sci. 21, 1266. 10.3390/ijms21041266 32070029 PMC7072933

[B28] Finch C. E. Tower J. (2014). Sex-specific aging in flies, worms, and missing great-granddads. Cell 156, 398–399. 10.1016/j.cell.2014.01.028 24485449 PMC3988832

[B29] Gao C. Fang L. Zhang H. Zhang W. S. Li X. O. Du S. Y. (2020). Metformin induces autophagy via the AMPK-mTOR signaling pathway in human hepatocellular carcinoma cells. Cancer Manag. Res. 12, 5803–5811. 10.2147/CMAR.S257966 32765083 PMC7371564

[B30] Gao D. Asghar S. Hu R. Chen S. Niu R. Liu J. (2023). Recent advances in diverse nanosystems for nitric oxide delivery in cancer therapy. Acta Pharm. Sin. B 13, 1498–1521. 10.1016/j.apsb.2022.11.016 37139410 PMC10149905

[B31] Gao Y. Yu X. F. Chen T. (2021). Human endogenous retroviruses in cancer: expression, regulation and function (Review). Oncol. Lett. 21, 121. 10.3892/ol.2020.12382 33552242 PMC7798031

[B32] Grani G. Ciotti L. Del Gatto V. Montesano T. Biffoni M. Giacomelli L. (2023). The legacy of the COVID-19 pandemics for thyroid cancer patients: towards the application of clinical practice recommendations. Endocrine 79, 45–48. 10.1007/s12020-022-03132-6 35857273 PMC9298162

[B33] Grimaldi C. Bleiberg H. Gay F. Messner M. Rougier P. Kok T. C. (1998). Evaluation of antiandrogen therapy in unresectable hepatocellular carcinoma: results of a European Organization for Research and Treatment of Cancer multicentric double-blind trial. J. Clin. Oncol. 16, 411–417. 10.1200/JCO.1998.16.2.411 9469323

[B34] Guo Y. Wu G. Yi J. Yang Q. Jiang W. Lin S. (2021). Anti-hepatocellular carcinoma effect and molecular mechanism of the estrogen signaling pathway. Front. Oncol. 11, 763539. 10.3389/fonc.2021.763539 35096574 PMC8789654

[B35] Hassan M. M. Botrus G. Abdel-Wahab R. Wolff R. A. Li D. Tweardy D. (2017). Estrogen replacement reduces risk and increases survival times of women with hepatocellular carcinoma. Clin. Gastroenterol. Hepatol. 15, 1791–1799. 10.1016/j.cgh.2017.05.036 28579181 PMC5901750

[B36] Herson P. S. Bombardier C. G. Parker S. M. Shimizu T. Klawitter J. Klawitter J. (2013). Experimental pediatric arterial ischemic stroke model reveals sex-specific estrogen signaling. Stroke 44, 759–763. 10.1161/STROKEAHA.112.675124 23349190 PMC3930081

[B37] Hill A. L. Khan M. Kiani A. Z. Lindemann J. D. Vachharajani N. Doyle M. B. (2023). Global liver transplantation: emerging trends and ethical challenges. Langenbecks Arch. Surg. 408, 418. 10.1007/s00423-023-03144-4 37875764

[B38] Hou J. Xu J. Jiang R. Wang Y. Chen C. Deng L. (2013). Estrogen-sensitive PTPRO expression represses hepatocellular carcinoma progression by control of STAT3. Hepatology 57, 678–688. 10.1002/hep.25980 22821478

[B39] Huillet M. Lasserre F. Gratacap M. P. Engelmann B. Bruse J. Polizzi A. (2024). Pharmacological activation of constitutive androstane receptor induces female-specific modulation of hepatic metabolism. JHEP Rep. 6, 100930. 10.1016/j.jhepr.2023.100930 38149074 PMC10749885

[B40] Izquierdo A. G. Carreira M. C. Rodriguez-Carnero G. Perez-Lois R. Seoane L. M. Casanueva F. F. (2022). Gender dimorphism in hepatic carcinogenesis-related gene expression associated with obesity as a low-grade chronic inflammatory disease. Int. J. Mol. Sci. 23, 15002. 10.3390/ijms232315002 36499327 PMC9739425

[B41] Jiang X. B. Ke C. Zhang G. H. Zhang X. H. Sai K. Chen Z. P. (2012). Brain metastases from hepatocellular carcinoma: clinical features and prognostic factors. BMC Cancer 12, 49. 10.1186/1471-2407-12-49 22292912 PMC3297522

[B42] Jiang Y. Han Q. J. Zhang J. (2019). Hepatocellular carcinoma: mechanisms of progression and immunotherapy. World J. Gastroenterol. 25, 3151–3167. 10.3748/wjg.v25.i25.3151 31333308 PMC6626719

[B43] KASL (2012). KASL clinical practice Guidelines: management of chronic hepatitis B. Clin. Mol. Hepatol. 18, 109–162. 10.3350/cmh.2012.18.2.109 22893865 PMC3415874

[B44] Kamimura K. Yokoo T. Abe H. Sakai N. Nagoya T. Kobayashi Y. (2020). Effect of diphtheria toxin-based gene therapy for hepatocellular carcinoma. Cancers (Basel) 12, 472. 10.3390/cancers12020472 32085552 PMC7072394

[B45] Kardashian A. Serper M. Terrault N. Nephew L. D. (2023). Health disparities in chronic liver disease. Hepatology 77, 1382–1403. 10.1002/hep.32743 35993341 PMC10026975

[B46] Karnam R. S. Chen S. Xu W. Chen C. Elangainesan P. Ghanekar A. (2021). Sex disparity in liver transplant and access to living donation. JAMA Surg. 156, 1010–1017. 10.1001/jamasurg.2021.3586 34406347 PMC8374729

[B47] Katleba K. D. Ghosh P. M. Mudryj M. (2023). Beyond prostate cancer: an androgen receptor splice variant expression in multiple malignancies, non-cancer pathologies, and development. Biomedicines 11, 2215. 10.3390/biomedicines11082215 37626712 PMC10452427

[B48] Ke Y. Zu S. Chen L. Liu M. Yang H. Wang F. (2022). Combination of estrogen receptor alpha and histological type helps to predict lymph node metastasis in patients with stage IA2 to IIA2 cervical cancer. Cancer Manag. Res. 14, 317–325. 10.2147/CMAR.S343518 35115830 PMC8802323

[B49] Kim W. R. Mannalithara A. Heimbach J. K. Kamath P. S. Asrani S. K. Biggins S. W. (2021). MELD 3.0: the model for end-stage liver disease updated for the modern era. Gastroenterology 161, 1887–1895.e4. 10.1053/j.gastro.2021.08.050 34481845 PMC8608337

[B50] Krolick K. N. Shi H. (2022). Estrogenic action in stress-induced neuroendocrine regulation of energy homeostasis. Cells 11, 879. 10.3390/cells11050879 35269500 PMC8909319

[B51] Kuwano A. Yada M. Narutomi F. Nagasawa S. Tanaka K. Kurosaka K. (2022). Therapeutic efficacy of atezolizumab plus bevacizumab for hepatocellular carcinoma with WNT/β-catenin signal activation. Oncol. Lett. 24, 216. 10.3892/ol.2022.13337 35720502 PMC9178725

[B52] Lee C. W. Yu M. C. Wang C. C. Lee W. C. Tsai H. I. Kuan F. C. (2021). Liver resection for hepatocellular carcinoma larger than 10 cm: a multi-institution long-term observational study. World J. Gastrointest. Surg. 13, 476–492. 10.4240/wjgs.v13.i5.476 34122737 PMC8167847

[B53] Li H. Guo L. Su K. Li C. Jiang Y. Wang P. (2023b). Construction and validation of tace therapeutic efficacy by alr score and nomogram: a large, multicenter study. J. Hepatocell. Carcinoma 10, 1009–1017. 10.2147/JHC.S414926 37405321 PMC10317537

[B54] Li H. Wu Z. Chen J. Su K. Guo L. Xu K. (2023a). External radiotherapy combined with sorafenib has better efficacy in unresectable hepatocellular carcinoma: a systematic review and meta-analysis. Clin. Exp. Med. 23, 1537–1549. 10.1007/s10238-022-00972-4 36495367 PMC10460724

[B55] Li Y. Yang D. Wang Y. Li Z. Zhu C. (2019). Co-delivery doxorubicin and silybin for anti-hepatoma via enhanced oral hepatic-targeted efficiency. Int. J. Nanomedicine 14, 301–315. 10.2147/IJN.S187888 30643408 PMC6314320

[B56] Li Y. Q. Wong C. S. (2018). Effects of p21 on adult hippocampal neuronal development after irradiation. Cell Death Discov. 4, 15. 10.1038/s41420-018-0081-2 PMC6131552 30210818

[B57] Li Z. Lan L. Zhou Y. Li R. Chavin K. D. Xu H. (2023c). Developing deep learning-based strategies to predict the risk of hepatocellular carcinoma among patients with nonalcoholic fatty liver disease from electronic health records. medRxiv. 10.1016/j.jbi.2024.104626 38521180

[B58] Lim S. Kaldis P. (2013). Cdks, cyclins and CKIs: roles beyond cell cycle regulation. Development 140, 3079–3093. 10.1242/dev.091744 23861057

[B59] Liu S. Wang R. Lou Y. Liu J. (2020). Uncovering the mechanism of the effects of pien-tze-huang on liver cancer using network Pharmacology and molecular docking. Evid. Based Complement. Altern. Med. 2020, 4863015. 10.1155/2020/4863015 PMC7492898 32963562

[B60] Liu Z. Song C. Suo C. Fan H. Zhang T. Jin L. (2022). Alcohol consumption and hepatocellular carcinoma: novel insights from a prospective cohort study and nonlinear Mendelian randomization analysis. BMC Med. 20, 413. 10.1186/s12916-022-02622-8 36303185 PMC9615332

[B61] LoMauro A. Aliverti A. (2021). Sex and gender in respiratory physiology. Eur. Respir. Rev. 30, 210038. 10.1183/16000617.0038-2021 34750114 PMC9488190

[B62] Ma W. L. Hsu C. L. Yeh C. C. Wu M. H. Huang C. K. Jeng L. B. (2012). Hepatic androgen receptor suppresses hepatocellular carcinoma metastasis through modulation of cell migration and anoikis. Hepatology 56, 176–185. 10.1002/hep.25644 22318717 PMC3673306

[B63] Madhu Krishna B. Chaudhary S. Mishra D. R. Naik S. K. Suklabaidya S. Adhya A. K. (2018). Estrogen receptor α dependent regulation of estrogen related receptor β and its role in cell cycle in breast cancer. BMC Cancer 18, 607. 10.1186/s12885-018-4528-x 29843638 PMC5975398

[B64] Mandalà M. (2020). Influence of estrogens on uterine vascular adaptation in normal and preeclamptic pregnancies. Int. J. Mol. Sci. 21, 2592. 10.3390/ijms21072592 32276444 PMC7177259

[B65] Marker P. C. Unterberger C. J. Swanson S. M. (2023). GH-dependent growth of experimentally induced carcinomas *in vivo* . Endocr. Relat. Cancer 30, e220403. 10.1530/ERC-22-0403 36826838 PMC10140676

[B66] Martínez Cerezo F. J. Tomás A. Donoso L. Enríquez J. Guarner C. Balanzó J. (1994). Controlled trial of tamoxifen in patients with advanced hepatocellular carcinoma. J. Hepatol. 20, 702–706. 10.1016/s0168-8278(05)80138-2 7930468

[B67] McGlynn K. A. Petrick J. L. El-Serag H. B. (2021). Epidemiology of hepatocellular carcinoma. Hepatology 73 (Suppl. 1), 4–13. 10.1002/hep.31288 PMC7577946 32319693

[B68] Memaj P. Ouzerara Z. Jornayvaz F. R. (2023). Role of oxidative stress and carcinoembryonic antigen-related cell adhesion molecule 1 in nonalcoholic fatty liver disease. Int. J. Mol. Sci. 24, 11271. 10.3390/ijms241411271 37511031 PMC10379080

[B69] Meng X. Liu X. (2022). Therapeutic value of estrogen receptor α in hepatocellular carcinoma based on molecular mechanisms. J. Clin. Transl. Hepatol. 10, 140–146. 10.14218/JCTH.2021.00224 35233383 PMC8845150

[B70] Miller L. E. (2018). Methylsulfonylmethane decreases inflammatory response to tumor necrosis factor-α in cardiac cells. Am. J. Cardiovasc Dis. 8, 31–38. 30038844 PMC6055070

[B71] Milman N. Kirchhoff M. (1996). Relationship between serum ferritin, alcohol intake, and social status in 2235 Danish men and women. Ann. Hematol. 72, 145–151. 10.1007/s002770050153 8766257

[B72] Mindikoglu A. L. Emre S. H. Magder L. S. (2013). Impact of estimated liver volume and liver weight on gender disparity in liver transplantation. Liver Transpl. 19, 89–95. 10.1002/lt.23553 23008117 PMC3535518

[B73] Mitchell T. De Miguel C. Gohar E. Y. (2020). Sex differences in redox homeostasis in renal disease. Redox Biol. 31, 101489. 10.1016/j.redox.2020.101489 32197946 PMC7212488

[B74] Mousavi S. F. Moosavy S. H. Alavian S. M. Eghbali H. Mahboobi H. (2013). Distribution of hepatitis C virus genotypes among patients with hepatitis C virus infection in hormozgan, Iran. Hepat. Mon. 13, e14324. 10.5812/hepatmon.14324 24403914 PMC3877657

[B75] Murgia F. Giagnoni F. Lorefice L. Caria P. Dettori T. D'Alterio M. N. (2022). Sex hormones as key modulators of the immune response in multiple sclerosis: a review. Biomedicines 10, 3107. 10.3390/biomedicines10123107 36551863 PMC9775368

[B76] Nan Y. Xu X. Gao Y. Wang R. Li W. Yang M. (2021). Consensus on the secondary prevention of primary liver cancer. Hepatol. Int. 15, 1289–1300. 10.1007/s12072-021-10259-7 34846705 PMC8712303

[B77] Nasr P. von Seth E. Mayerhofer R. Ndegwa N. Ludvigsson J. F. Hagström H. (2023). Incidence, prevalence and mortality of chronic liver diseases in Sweden between 2005 and 2019. Eur. J. Epidemiol. 38, 973–984. 10.1007/s10654-023-01028-x 37490175 PMC10501948

[B78] Okoronkwo N. Wang Y. Pitchumoni C. Koneru B. Pyrsopoulos N. (2017). Improved outcomes following hepatocellular carcinoma (HCC) diagnosis in patients screened for HCC in a large academic liver center versus patients identified in the community. J. Clin. Transl. Hepatol. 5, 31–34. 10.14218/JCTH.2016.00051 28507924 PMC5411354

[B79] Pan Y. Sun C. Huang M. Liu Y. Qi F. Liu L. (2014). A genetic variant in pseudogene E2F3P1 contributes to prognosis of hepatocellular carcinoma. J. Biomed. Res. 28, 194–200. 10.7555/JBR.28.20140052 25013402 PMC4085556

[B80] Pok S. Barn V. A. Wong H. J. Blackburn A. C. Board P. Farrell G. C. (2016). Testosterone regulation of cyclin E kinase: a key factor in determining gender differences in hepatocarcinogenesis. J. Gastroenterol. Hepatol. 31, 1210–1219. 10.1111/jgh.13232 26574916

[B81] Poorolajal J. Majdzadeh R. (2009). Prevalence of chronic hepatitis B infection in Iran: a review article. J. Res. Med. Sci. 14, 249–258. 21772891 PMC3129112

[B82] Puri N. DeBeck K. Feng C. Kerr T. Rieb L. Wood E. (2014). Gender influences on hepatitis C incidence among street youth in a Canadian setting. J. Adolesc. Health 55, 830–834. 10.1016/j.jadohealth.2014.07.006 25240449 PMC4254041

[B83] Qi Y. Liu Y. Yu B. Hu Y. Zhang N. Zheng Y. (2020). A lactose-derived CRISPR/Cas9 delivery system for efficient genome editing *in vivo* to treat orthotopic hepatocellular carcinoma. Adv. Sci. (Weinh) 7, 2001424. 10.1002/advs.202001424 32995132 PMC7507475

[B84] Qiao Y. Wang X. M. Mannan R. Pitchiaya S. Zhang Y. Wotring J. W. (2021). Targeting transcriptional regulation of SARS-CoV-2 entry factors ACE2 and TMPRSS2. Proc. Natl. Acad. Sci. U. S. A. 118, e2021450118. 10.1073/pnas.2021450118 33310900 PMC7817128

[B85] Rehman H. U. Masson E. A. (2005). Neuroendocrinology of female aging. Gend. Med. 2, 41–56. 10.1016/s1550-8579(05)80008-7 16115597

[B86] Sanaei M. J. Razi S. Pourbagheri-Sigaroodi A. Bashash D. (2022). The PI3K/Akt/mTOR pathway in lung cancer; oncogenic alterations, therapeutic opportunities, challenges, and a glance at the application of nanoparticles. Transl. Oncol. 18, 101364. 10.1016/j.tranon.2022.101364 35168143 PMC8850794

[B87] Sayaf K. Gabbia D. Russo F. P. De Martin S. (2022). The role of sex in acute and chronic liver damage. Int. J. Mol. Sci. 23, 10654. 10.3390/ijms231810654 36142565 PMC9505609

[B88] Shi C. Y. Phang T. W. Wee A. Ngoi S. S. Lin Y. Li B. (1995). Mutations of the tumour suppressor gene p53 in colorectal and hepatocellular carcinomas. Ann. Acad. Med. Singap 24, 204–210. 7653961

[B89] Singh S. Hoque S. Zekry A. Sowmya A. (2023). Radiological diagnosis of chronic liver disease and hepatocellular carcinoma: a review. J. Med. Syst. 47, 73. 10.1007/s10916-023-01968-7 37432493 PMC10335966

[B90] Singhal P. C. Schlondorff D. (1987). Hyperosmolal state associated with rhabdomyolysis. Nephron 47, 202–204. 10.1159/000184492 3683689

[B91] Song H. Yu Z. Sun X. Feng J. Yu Q. Khan H. (2018). Androgen receptor drives hepatocellular carcinogenesis by activating enhancer of zeste homolog 2-mediated Wnt/β-catenin signaling. EBioMedicine 35, 155–166. 10.1016/j.ebiom.2018.08.043 30150059 PMC6156715

[B92] Su J. Liu X. Zhao X. Ma H. Jiang Y. Wang X. (2024). Curcumin inhibits the growth of hepatocellular carcinoma via the MARCH1-mediated modulation of JAK2/STAT3 signaling. Recent Pat. Anticancer Drug Discov. 19. 10.2174/0115748928261490231124055059 38243928

[B93] Su K. Guo L. Ma W. Wang J. Xie Y. Rao M. (2022b). PD-1 inhibitors plus anti-angiogenic therapy with or without intensity-modulated radiotherapy for advanced hepatocellular carcinoma: a propensity score matching study. Front. Immunol. 13, 972503. 10.3389/fimmu.2022.972503 36211350 PMC9539675

[B94] Su K. Liu Y. Wang P. He K. Wang F. Chi H. (2022a). Heat-shock protein 90α is a potential prognostic and predictive biomarker in hepatocellular carcinoma: a large-scale and multicenter study. Hepatol. Int. 16, 1208–1219. 10.1007/s12072-022-10391-y 35972640 PMC9525341

[B95] Su K. Shen Q. Tong J. Gu T. Xu K. Li H. (2023b). Construction and validation of a nomogram for HBV-related hepatocellular carcinoma: a large, multicenter study. Ann. Hepatol. 28, 101109. 10.1016/j.aohep.2023.101109 37100384

[B96] Su K. Wang F. Li X. Chi H. Zhang J. He K. (2023a). Effect of external beam radiation therapy versus transcatheter arterial chemoembolization for non-diffuse hepatocellular carcinoma (≥5 cm): a multicenter experience over a ten-year period. Front. Immunol. 14, 1265959. 10.3389/fimmu.2023.1265959 37818373 PMC10560878

[B97] Sun E. J. Wankell M. Palamuthusingam P. McFarlane C. Hebbard L. (2021). Targeting the PI3K/Akt/mTOR pathway in hepatocellular carcinoma. Biomedicines 9, 1639. 10.3390/biomedicines9111639 34829868 PMC8615614

[B98] Tang N. Dou X. You X. Li Y. Li X. Liu G. (2021). Androgen receptors act as a tumor suppressor gene to suppress hepatocellular carcinoma cells progression via miR-122-5p/RABL6 signaling. Front. Oncol. 11, 756779. 10.3389/fonc.2021.756779 34745992 PMC8564478

[B99] Tian L. Y. Smit D. J. Jücker M. (2023). The role of PI3K/AKT/mTOR signaling in hepatocellular carcinoma metabolism. Int. J. Mol. Sci. 24, 2652. 10.3390/ijms24032652 36768977 PMC9916527

[B100] Tsang D. P. Wu W. K. Kang W. Lee Y. Y. Wu F. Yu Z. (2016). Yin Yang 1-mediated epigenetic silencing of tumour-suppressive microRNAs activates nuclear factor-κB in hepatocellular carcinoma. J. Pathol. 238, 651–664. 10.1002/path.4688 26800240

[B101] Villa E. Dugani A. Fantoni E. Camellini L. Buttafoco P. Grottola A. (1996). Type of estrogen receptor determines response to antiestrogen therapy. Cancer Res. 56, 3883–3885. 8752151

[B102] Wang C. H. Lin R. C. Hsu H. Y. Tseng Y. T. (2022). Hormone replacement therapy is associated with reduced hepatocellular carcinoma risk and improved survival in postmenopausal women with hepatitis B: a nationwide long-term population-based cohort study. PLoS One 17, e0271790. 10.1371/journal.pone.0271790 35862398 PMC9302748

[B103] Wang H. Zheng Y. Huang J. Li J. (2021). Mitophagy in antiviral immunity. Front. Cell Dev. Biol. 9, 723108. 10.3389/fcell.2021.723108 34540840 PMC8446632

[B104] Wei K. R. Yu X. Zheng R. S. Peng X. B. Zhang S. W. Ji M. F. (2014). Incidence and mortality of liver cancer in China, 2010. Chin. J. Cancer 33, 388–394. 10.5732/cjc.014.10088 25104174 PMC4135368

[B105] Wen S. Niu Y. Lee S. O. Chang C. (2014). Androgen receptor (AR) positive vs negative roles in prostate cancer cell deaths including apoptosis, anoikis, entosis, necrosis and autophagic cell death. Cancer Treat. Rev. 40, 31–40. 10.1016/j.ctrv.2013.07.008 23993415 PMC3833078

[B106] Williams G. M. Iatropoulos M. J. Karlsson S. (1997). Initiating activity of the anti-estrogen tamoxifen, but not toremifene in rat liver. Carcinogenesis 18, 2247–2253. 10.1093/carcin/18.11.2247 9395228

[B107] Wojnarowski K. Cholewińska P. Palić D. Bednarska M. Jarosz M. Wiśniewska I. (2022). Estrogen receptors mediated negative effects of estrogens and xenoestrogens in teleost fishes-review. Int. J. Mol. Sci. 23, 2605. 10.3390/ijms23052605 35269746 PMC8910684

[B108] Wu C. T. Chen W. C. Lin P. Y. Liao S. K. Chen M. F. (2009). Androgen deprivation modulates the inflammatory response induced by irradiation. BMC Cancer 9, 92. 10.1186/1471-2407-9-92 19320990 PMC2667536

[B109] Wu J. Zhang J. Shen B. Yin K. Xu J. Gao W. (2015). Long noncoding RNA lncTCF7, induced by IL-6/STAT3 transactivation, promotes hepatocellular carcinoma aggressiveness through epithelial-mesenchymal transition. J. Exp. Clin. Cancer Res. 34, 116. 10.1186/s13046-015-0229-3 26452542 PMC4600266

[B110] Wu M. Lou S. (2023). Deciphering the influence of estradiol and estrogen receptors on cognitive function: a bibliometric analysis and emerging research trends. Med. Sci. Monit. 29, e939676. 10.12659/MSM.939676 37300249 PMC10266109

[B111] Xu D. Wang X. Yan S. Yin Y. Hou J. Wang X. (2014). Interaction of PTPRO and TLR4 signaling in hepatocellular carcinoma. Tumour Biol. 35, 10267–10273. 10.1007/s13277-014-2302-5 25034527

[B112] Yan W. Cheng L. Zhang D. (2020). Ultrasound-targeted microbubble destruction mediated si-CyclinD1 inhibits the development of hepatocellular carcinoma via suppression of PI3K/AKT signaling pathway. Cancer Manag. Res. 12, 10829–10839. 10.2147/CMAR.S263590 33149688 PMC7605614

[B113] Yang X. Schadt E. E. Wang S. Wang H. Arnold A. P. Ingram-Drake L. (2006). Tissue-specific expression and regulation of sexually dimorphic genes in mice. Genome Res. 16, 995–1004. 10.1101/gr.5217506 16825664 PMC1524872

[B114] Yu P. Duan X. Cheng Y. Liu C. Chen Y. Liu W. (2017). Androgen-independent LNCaP cells are a subline of LNCaP cells with a more aggressive phenotype and androgen suppresses their growth by inducing cell cycle arrest at the G1 phase. Int. J. Mol. Med. 40, 1426–1434. 10.3892/ijmm.2017.3125 28901378 PMC5627872

[B115] Zhang H. Li X. X. Yang Y. Zhang Y. Wang H. Y. Zheng X. F. S. (2018). Significance and mechanism of androgen receptor overexpression and androgen receptor/mechanistic target of rapamycin cross-talk in hepatocellular carcinoma. Hepatology 67, 2271–2286. 10.1002/hep.29715 29220539 PMC6106789

[B116] Zhang J. Dong K. Zhang X. Li C. Yu J. Wang W. (2023b). Characteristics of lactate metabolism phenotype in hepatocellular carcinoma. Sci. Rep. 13, 19674. 10.1038/s41598-023-47065-0 37952028 PMC10640573

[B117] Zhang S. Jiang C. Jiang L. Chen H. Huang J. Gao X. (2023a). Construction of a diagnostic model for hepatitis B-related hepatocellular carcinoma using machine learning and artificial neural networks and revealing the correlation by immunoassay. Tumour Virus Res. 16, 200271. 10.1016/j.tvr.2023.200271 37774952 PMC10638043

[B118] Zhang W. Liu F. Huang J. Guo X. Dong W. Wei S. (2020). Effect of menopausal status on the survival and recurrence of sex-classified hepatocellular carcinoma after liver resection: a case-matched study with propensity score matching. Aging (Albany NY) 12, 25895–25915. 10.18632/aging.202155 33232278 PMC7803575

[B119] Zhang Y. Zhang X. Li W. Du Y. Hu W. Zhao J. (2022). Biomarkers and risk factors for the early prediction of immune-related adverse events: a review. Hum. Vaccin Immunother. 18, 2018894. 10.1080/21645515.2021.2018894 35108160 PMC8986173

[B120] Zhao P. Malik S. Xing S. (2021). Epigenetic mechanisms involved in HCV-induced hepatocellular carcinoma (HCC). Front. Oncol. 11, 677926. 10.3389/fonc.2021.677926 34336665 PMC8320331

[B121] Zhu H. Chen Y. Zhang J. Qian C. Qiu W. Shen H. (2020). Knockdown of TRIM37 promotes apoptosis and suppresses tumor growth in gastric cancer by inactivation of the ERK1/2 pathway. Onco Targets Ther. 13, 5479–5491. 10.2147/OTT.S233906 32606764 PMC7297455

