# Supplementary material for: Exploiting gender-based biomarkers and drug targets: advancing personalized therapeutic strategies in hepatocellular carcinoma
Source: Front Pharmacol. 2024 Jun 20;15:1433540. doi: 10.3389/fphar.2024.1433540 (PMC11222576; doi:10.3389/fphar.2024.1433540)
Supplement: Supplementary file 1 [file Table1.DOCX]

| Forms | Factors | Regimes | Effects | Position | References |
| --- | --- | --- | --- | --- | --- |
|  |  |  |  |  |  |
| Estrogens and their receptors | PTPRO | Cause a decrease in the activity of the transcription factor STAT3 | Inhibit HCC cell proliferation | Nucleus, cytoplasm | Estrogen-sensitive PTPRO expression represses hepatocellular carcinoma progression by control of STAT3  [65] |
|  | PPARα | Reduce the transcription of the PPARα gene and further regulates ACO, cell cycle protein Dl, P27, etc. | Block cancer cell proliferation and promote apoptosis | Nucleus, cytoplasm | E2/ERβ Inhibits PPARα to Regulate Cell-Proliferation and Enhance Apoptosis in Hep3B-Hepatocellular Carcinoma  [66] |
|  | P53 protein | Avoid abnormal cell cycle arrest and apoptosis and enhance DNA damage repair | Reduce tumorigenesis | Nucleus, cytoplasm | Epigenetic Mechanisms Involved in HCV-Induced Hepatocellular Carcinoma (HCC)  [67] |
|  | mTOR | Activate PI3K-mTOR signaling | Promote liver cancer cell survival and proliferation | Nucleus, cytoplasm | Estrogen Activation of G-Protein-Coupled Estrogen Receptor 1 Regulates Phosphoinositide 3-Kinase and mTOR Signaling to Promote Liver Growth in Zebrafish and Proliferation of Human Hepatocytes  [68] |
| Estrogen receptor complexes and transcription factors | The inhibitory factor NF-κB | Inhibit activation of the IL-6/STAT3 pathway | Enhance HCC cell migration and invasion, proliferation and viability | Cytoplasm, cell membrane | Anti-Hepatocellular Carcinoma Effect and Molecular Mechanism of the Estrogen Signaling Pathway  [69] |
| Androgens and their receptors | EZH2 | Silence Wnt signaling inhibitors, thereby activating Wnt/β-cyclin signaling | Promote proliferation of liver tumor cells | Nucleus, cytoplasm | Androgen receptor drives hepatocellular carcinogenesis by activating enhancer of zeste homolog 2-mediated Wnt/β-catenin signaling  [70] |
|  | the transcription factors hypoxia-inducible factor 2α (HIF-2α) | AR-regulated HIF-2α inhibits c-Myc expression | Inhibit the proliferation of hepatocellular carcinoma cells | Nucleus, cytoplasm | miR-135b-5p Suppresses Androgen Receptor-Enhanced Hepatocellular Carcinoma Cell Proliferation via Regulating the HIF-2α/c-Myc/P27 Signals in vitro  [61] |
|  | mTOR | Activate the PI3K/AKT/mTOR pathway, which in turn may upregulate integrin β1 expression and enhance the phosphorylation level of AKT | Enhance cell adhesion | Nucleus, cytoplasm | Targeting the PI3K/Akt/mTOR Pathway in Hepatocellular Carcinoma  [71] |
